# Finger volume pulse waveforms facilitate reliable assessment of heart rate variability, but not blood pressure variability or baroreflex function

**DOI:** 10.1186/1471-2261-14-180

**Published:** 2014-12-09

**Authors:** Jonathan R Linder, Harald M Stauss, Holly Gindes, Gary L Pierce, Nicholas H Von Bergen, William G Haynes, Jess G Fiedorowicz

**Affiliations:** Department of Internal Medicine, The University of Iowa, Iowa City, IA 52242 USA; Department of Pediatrics, The University of Iowa, Iowa City, IA 52242 USA; Department of Psychiatry, Roy J. and Lucille A. Carver College of Medicine, The University of Iowa, Iowa City, IA 52242 USA; Institute for Clinical and Translational Science, The University of Iowa, Iowa City, IA 52242 USA; Department of Health and Human Physiology, College of Liberal Arts & Sciences, The University of Iowa, Iowa City, IA 52242 USA; Department of Epidemiology, College of Public Health, The University of Iowa, Iowa City, IA 52242 USA; College of Pharmacy, The University of Iowa, Iowa City, IA 52242 USA; 200 Hawkins Drive W278GH, Iowa City, IA 52242-1057 USA

**Keywords:** EndoPAT, Finometer Pro, Cardiovascular function, Device validation

## Abstract

**Background:**

We sought to determine whether heart rate variability (HRV), blood pressure (BP) variability, and baroreceptor-heart rate reflex sensitivity can be reliably assessed using finger volume pulse waveforms obtained from the commercially available EndoPAT device.

**Methods:**

Non-invasive BP (Finometer Pro as a non-invasive standard) and finger volume (EndoPAT) waveforms were recorded in 65 adults (37 ± 14 years; 60% female) and systolic BP and heart rate (HR) time series were derived after calibrating the EndoPAT signal based on systolic and diastolic BP values obtained by a sphygomomanometer. Transfer function analyses were performed to test for coherence between systolic BP and HR time series derived from the Finometer and EndoPAT devices. Time-domain HRV parameters, frequency domain HR and systolic BP variability parameters, and baroreflex sensitivity (sequence technique) were computed from Finometer- and EndoPAT-derived time series and intraclass correlation coefficients (ICC) were calculated.

**Results:**

Squared coherence between systolic BP time series derived from the Finometer and EndoPAT devices was low, suggesting poor correlation. In contrast, squared coherence between HR time series derived from the two devices was excellent [High Frequency (HF) = 0.80, Low Frequency (LF) = 0.81], with gain values close to 1.0. ICC values for time- and frequency-domain HRV parameters were excellent (>0.9 except for relative HF HRV, which was 0.77), while ICC values for frequency-domain BP variability parameters and baroreceptor-HR reflex sensitivity were low.

**Conclusions:**

Finger volume pulse waveforms can be used to reliably assess both time-domain and frequency-domain HR variability. However, frequency domain BP variability parameters cannot be reliably assessed from finger volume pulse waveforms using the simple calibration technique used in this study.

## Background

Autonomic regulation of heart rate (HR) and blood pressure (BP) is commonly cited as an indicator of cardiovascular health in humans [1–4]. Time- and frequency-domain HR variability (HRV) provide reliable estimates of both sympathetic and parasympathetic modulation of cardiac function [2, 3, 5, 6], while low frequency BP variability (BPV) reflects sympathetic modulation of vascular tone [4, 7, 8]. Baroreceptor-HR reflex sensitivity is another common measure of cardio-autonomic health, providing insight into cardiovascular regulation by the baroreceptors [9, 10].

Time-domain HRV can be reliably assessed by deriving the standard deviation of NN intervals (SDNN) and the root-mean-square of successive differences (RMSSD) from normal sinus rhythm pulse intervals (NN). SDNN is the standard deviation of all NN intervals over a fixed amount of time, usually 5-minutes [1, 3]. NN intervals are the measured intervals between consecutive heartbeats originating from a sinus rhythm. SDNN is an indicator of both sympathetic and parasympathetic cardiac inputs, and is thus a representative estimate of *overall* autonomic regulation of HRV. RMSSD is the square root of the mean of the squared differences between successive NN intervals. RMSSD is considered to reflect mainly parasympathetic and less sympathetic modulation of cardiac function [1].

Spectral analysis can be used to decompose oscillatory components of HRV and BPV into high frequency (HF), low frequency (LF), and very low frequency (VLF) bands. For HRV, the HF range (0.15-0.4) is representative of respiration-linked parasympathetic cardiac modulation, LF (0.06-0.15) is considered to be indicative of both parasympathetic and sympathetic modulation, and VLF (0.02-0.06) is sometimes considered representative of sympathetic cardiac modulation, but its true physiologic/pathophysiologic role is unclear [1, 2, 7]. For BPV in humans, the HF (0.15-0.4) component is largely dependent on respiration-linked fluctuations in stroke volume possibly caused by respiratory mechanics. LF BPV (0.075-0.15) is mostly representative of sympathetic modulation of vascular tone, while VLF BPV (0.02-0.07) results from several mechanisms, including myogenic vascular control and endocrine factors (e.g., renin-angiotensin system) [4, 8, 11].

In this study, we sought to validate the measurements obtained from a commercially available EndoPAT device (Itamar Medical Ltd., Caesarea, Israel) using finger volume pulse waveforms to derive HRV and BPV parameters and to estimate baroreceptor-HR reflex sensitivity. BP waveforms obtained from a commercially available Finometer Pro device (Finapres Medical Systems, Amsterdam, The Netherlands) were used to validate the EndoPAT-derived HRV and BPV parameters and baroreceptor-HR reflex sensitivity. The EndoPAT device measures arterial pulsatile volume changes in the fingertips by plethysmography. This reportedly allows assessment of arterial dilator capability, suggested to be an indicator of overall cardiovascular health [12]. This technology has the potential to allow non-invasive measurement of HRV and BPV, however has yet to be validated for these uses. The EndoPAT device provides waveforms that potentially allow for reliable beat-by-beat HR and BP trends to be derived and subsequently used for HRV, BPV, and baroreflex analyses. The Finometer Pro, a non-invasive BP monitoring system, evaluates finger arterial BP measurements detected by an infrared photoplethysmograph and a finger pressure cuff. The Finometer Pro can be used as a non-invasive standard for measuring HRV and BPV in humans [13, 14]. The Finometer Pro and related machines have been validated in a wide variety of patient populations for HRV, BPV and baroreflex analyses [15–17].

We hypothesized that the EndoPAT finger volume pulse waveforms would provide reliable estimates for both time-domain and frequency-domain HRV. We also sought to determine whether these waveforms can be used to estimate BPV and/or baroreflex sensitivity after appropriate calibration.

## Methods

### Sample

The protocol was approved by the Institutional Review Board at the University of Iowa and was conducted in compliance with the Declaration of Helsinki. Participants were recruited using a University-wide email and a local print advertisement at the University of Iowa Hospitals and Clinics. Participants were excluded if they had significant hand injuries. All participants were asked to abstain from food, caffeine, and tobacco for two hours before each visit. We obtained informed consent from each participant, and a member of the research team assessed history of illness, recreational drug exposure, alcohol exposure, and tobacco exposure (pack per day*years). Alcohol exposure was assessed using the AUDIT-C screener [18], and participants were classified as either positive or negative for alcohol misuse. Men were classified under alcohol misuse following an AUDIT-C result of 4 or higher, while women were classified under alcohol misuse following an AUDIT-C result of 3 or higher.

### HRV, BPV, and baroreflex assessment

For all participants, BP waveforms were recorded non-invasively in the middle finger of the left hand for 30 minutes at a sampling rate of 200 Hz using the Finometer Pro device (non-invasive “gold standard”). Simultaneously, finger volume pulse waveforms were also recorded in the index fingers of both hands non-invasively using the EndoPAT device, sampling at a frequency of 128 Hz. Only the EndoPAT recordings for the left hand were used for subsequent analyses. The non-calibrated finger volume pulse waveforms were calibrated so that the average systolic peaks and diastolic troughs of the 30-minute waveforms corresponded to the systolic and diastolic BP values obtained using a sphygmomanometer at the beginning of the protocol. From these waveforms, systolic BP and heart rate were derived using the Analyzer module of the freely available HemoLab software (http://www.haraldstauss.com/HaraldStaussScientific/hemolab/default.html) on a beat-by-beat basis. HR and systolic BP were derived from the calibrated EndoPAT waveform. From this signal, beat-by-beat trends in heart rate and systolic BP were derived in the HemoLab software analyzer module. The Finometer provided an automatically derived trend for both HR and BP. No filters were used during data acquisition or subsequent data analysis.

Heart rate and systolic BP values affected by recording artifacts or cardiac arrhythmias were visually detected and replaced by interpolated values. The following analysis was performed using the Batch Processor module of the HemoLab software: Time-domain HRV parameters were calculated from the beat-by-beat HR time series using a stationary artifact free segment of 5-minute duration. For frequency-domain analysis, the beat-by-beat systolic BP and HR time series were interpolated (cubic spline) to obtain equidistant time series at 10 Hz sampling rate. Absolute and relative (absolute spectral power divided by total spectral power) spectral powers were calculated in the VLF (0.02-0.05 Hz) [2, 4], LF (0.05-0.15 Hz), and HF (0.15-0.50 Hz) bands by applying the fast Fourier transform (FFT, ~410 s long segments, 50% overlap) to the full 30 minute long time series. Time-domain analysis was limited to 5-minute segments, as recommended [1], because duration affects the size of the results in time-domain analysis. Results from frequency-domain analysis, on the other hand, are not affected by duration of the segments analyzed.

Baroreceptor-heart rate reflex sensitivity was calculated from the 30 minute Finometer-derived BP and EndoPAT-derived calibrated finger volume pulse waveforms using the sequence technique implemented in the Analyzer module of the HemoLab software. The entire recording was selected for analysis of each device, separately. Systolic BP was analyzed using the “*Calculate-Baroreflex*” function with an “R for inclusion” of 0.8. A delay of three heartbeats was selected between the beat-by-beat systolic BP and pulse interval values.

### Transfer function analysis

In order to assess the correlation between Finometer-derived and EndoPAT-derived time series, transfer function analysis was performed that provides the squared coherence and the gain between the two time series. The squared coherence provides values (between 0 and 1) similar to the R^2^ value of a correlation analysis for each frequency component of the two time series. The gain of the transfer function can be seen as a factor by which each frequency component of one time series is amplified (gain >1.0) or reduced (gain <1.0) compared to the other time series. Transfer function analysis was performed from the spline interpolated (10 Hz) systolic BP time series derived from the EndoPAT- and Finometer-derived waveforms. The same protocol was performed for the HR time series derived from both respective machines. The squared coherence function γ(q) and gain H(q) of the transfer functions were calculated on the basis of the autospectral density functions and the cross-spectrum of the input (Finometer-derived time series) and output (EndoPAT-derived) functions. This was done using the Batch processor module of the HemoLab software.

Subsequently, the maximum gain and coherence values for both the HF and LF spectral domains were calculated for each participant. This was done for the transfer functions calculated from both the HR and BP time series. Due to systematic detection of cardiac arrhythmias, four participants were excluded from the transfer function analysis.

### Statistical analyses

Data are presented as mean ± SD. Study data were collected and managed using REDCap (Research Electronic Data Capture) tools hosted at The University of Iowa Hospitals and Clinics. Data was merged using SAS 9.3 (SAS Institute Inc., Cary, NC) and descriptive statistics for the sample were compiled. Intraclass correlation coefficients between parameters derived from Finometer-(BP) and EndoPAT-derived (finger volume pulse) waveforms were calculated using SPSS Statistics 19.0 (IBM Corp. Armonk, NY). Bland-Altman plots were created using SigmaPlot 12.0 (Systat Software, San Jose, CA) to display agreement across the range of observed values.

## Results

Sociodemographic and clinical characteristics of our sample are outlined in Table 1. The sample had a mean (SD) age of 37 (14) and was 60% female.

**Table 1:** **Sociodemographic and clinical characteristics (N = 65)**

	Mean (SD)
Age	37 (14)
Pack years (Smoking)	
Entire sample	2.6 (7.6)
Smokers only	12.4 (12.5)
Body mass index (kg/m2)	27.9 (6.5)
Heart rate (bpm)	70.9 (13)
Systolic blood pressure (mmHg)	119.5 (13.0)
Diastolic blood pressure (mmHg)	74.9 (8.6)
Respiratory rate (min^-1^)	13.4 (3)
	N (%)
Female gender	39 (60%)
White, not hispanic	61 (93.8%)
Unmarried	32 (48%)
Unemployed	3 (4.6%)
Alcohol misuse	31 (47%)
History of tobacco use	14 (22%)
Heart attack	1 (1.5%)
High blood pressure	9 (13.6%)
Diabetes or high blood sugar	4 (6%)

Transfer function analysis (Table 2) revealed that squared coherence for systolic BP time series derived from the Finometer and EndoPAT devices was relatively low (<0.35) for the LF and HF bands. This finding indicates a poor correlation between systolic BP values derived from the Finometer and EndoPAT devices. As a result of the low squared coherence, we don’t feel that the gain values can be interpreted in a meaningful way.

**Table 2:** **Transfer function analysis between finometer- and EndoPAT-derived time series**

	Low frequency		High frequency	
Mean(SD)	Coherence	Gain	Coherence	Gain
**Systolic BP**	0.33 (0.13)	0.90 (0.45)	0.27 (0.08)	0.55 (0.34)
**HR**	0.81 (0.17)	0.94 (0.14)	0.80 (0.17)	1.03 (0.22)

In contrast to systolic BP, the squared coherence for the HR time series derived from the two devices was high (HF = 0.80, LF = 0.81), indicating excellent agreement between Finometer- and EndoPAT- derived HR time series. In addition, the gain ~1.0 indicates that the HR values correspond in absolute terms.

Results of our primary analysis are summarized in Table 3. Intraclass correlation coefficients (ICC) for time-domain HRV showed a strong association for both SDNN (0.99) and RMSSD (0.96). Frequency-domain HRV showed similarly strong ICCs for both absolute and relative measures of VLF, LF, and HF spectral powers (ranging from 0.77 to 0.97, Table 3). The frequency domain BPV analysis garnered much weaker ICCs than HRV analysis with ICC values ranging from 0.34 to 0.64 (Table 3).

**Table 3:** **Intraclass correlation coefficients for primary outcomes**

Measures	Intraclass correlation coefficients between EndoPAT and finometer		
		95% Confidence interval	
	ICC	Lower bound	Upper bound
SDNN	0.99	0.98	0.99
RMSSD	0.96	0.94	0.98
Absolute VLF of HRV	0.94	0.90	0.96
Relative VLF of HRV	0.94	0.90	0.96
Absolute LF of HRV	0.94	0.90	0.96
Relative LF of HRV	0.92	0.87	0.95
Absolute HF of HRV	0.97	0.95	0.98
Relative HF of HRV	0.77	0.65	0.86
Absolute VLF of BPV	0.34	0.11	0.54
Relative VLF of BPV	0.37	0.14	0.56
Absolute LV of BPV	0.43	0.20	0.61
Relative LF of BPV	0.64	0.47	0.77
Absolute HF of BPV	0.57	0.38	0.71
Relative HF of BPV	0.51	0.31	0.67
Baroreflex gain	0.27	0.02	0.49

Baroreceptor-heart rate reflex sensitivity values derived from the two waveforms were not well correlated (ICC = 0.27). From the EndoPAT-derived time series, only 4.5 (SD 4.0) baroreflex sequences were detected per 1000 heartbeats compared to 6.1 (8.0) sequences per 1000 heart beats detected in the Finometer-derived time series. However, this difference in number of sequences was not significant (Wilcoxon Signed Rank S = -211, p = 0.16). EndoPAT-derived time series tended to overestimate baroreflex gain relative to the Finometer-derived time series [mean (SD)] 23.9 (12.0) ms/mmHg vs. 15.7 (8.2) ms/mmHg and EndoPAT-derived baroreflex gains did not appear to reliably correlate to the Finometer-derived gains (ICC = 0.27, 95% C.I. 0.02-0.49).

**Figure 1:**
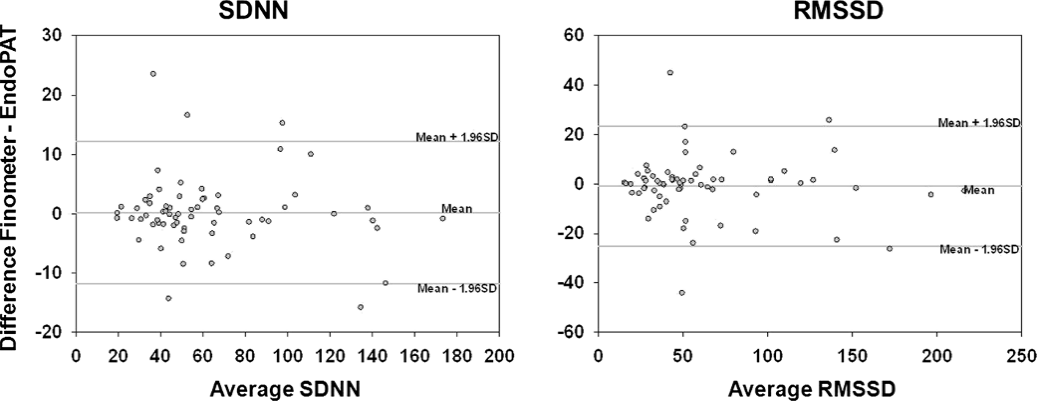
**Bland-Altman plots of time domain HRV parameters derived from EndoPAT and Finometer devices.** Bland-Altman comparison between EndoPAT- and Finometer-derived heart rate time series shows a high agreement between the two devices for the measurement of SDNN and RMSSD.

**Figure 2:**
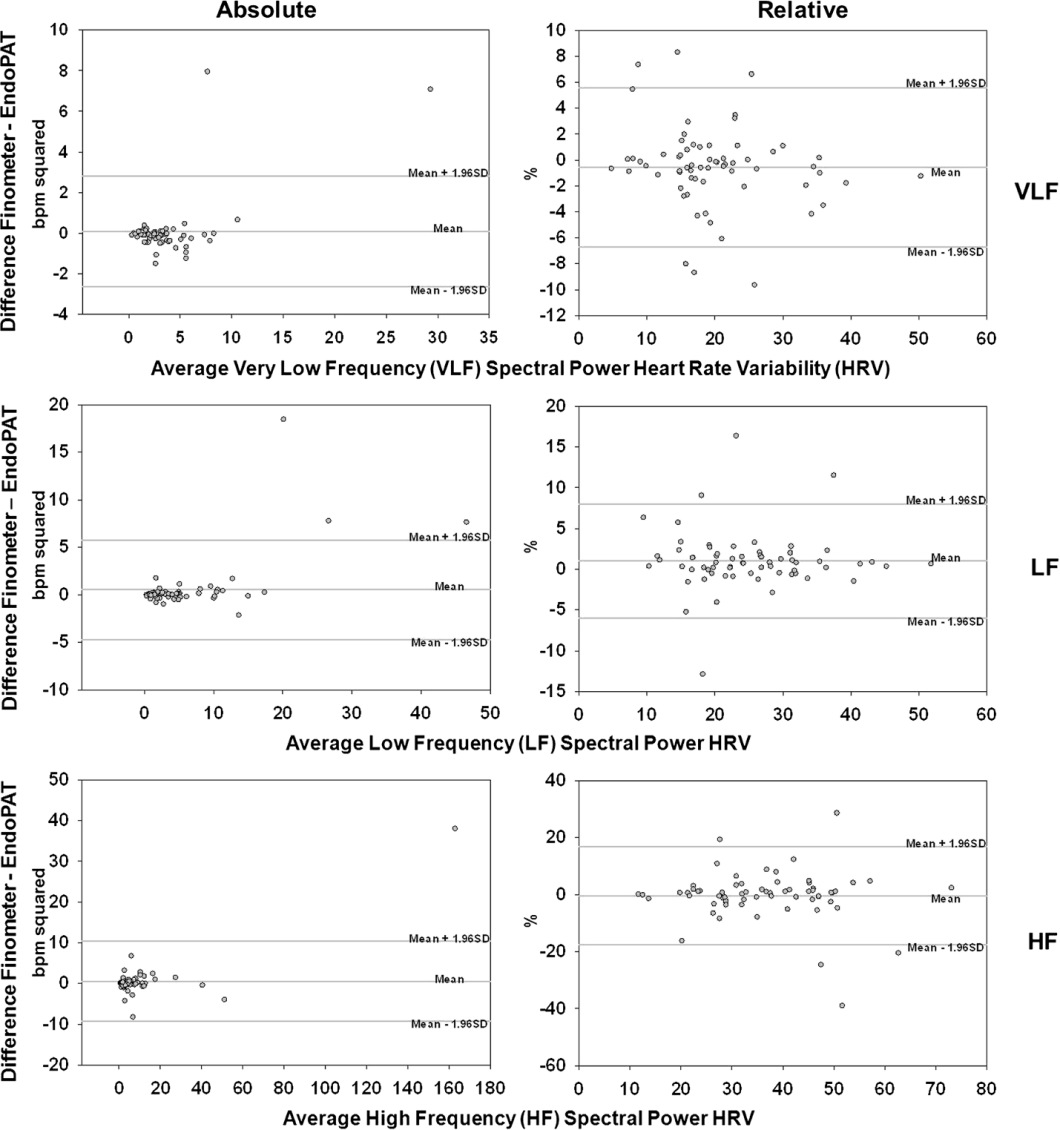
**Bland-Altman plots of frequency domain HRV parameters derived from EndoPAT and Finometer devices.** Bland-Altman comparison of frequency domain HRV parameters between EndoPAT- and Finometer-derived time series shows a high agreement between the two devices — across VLF, LF, and HF spectral ranges for both absolute and relative HRV.

**Figure 3:**
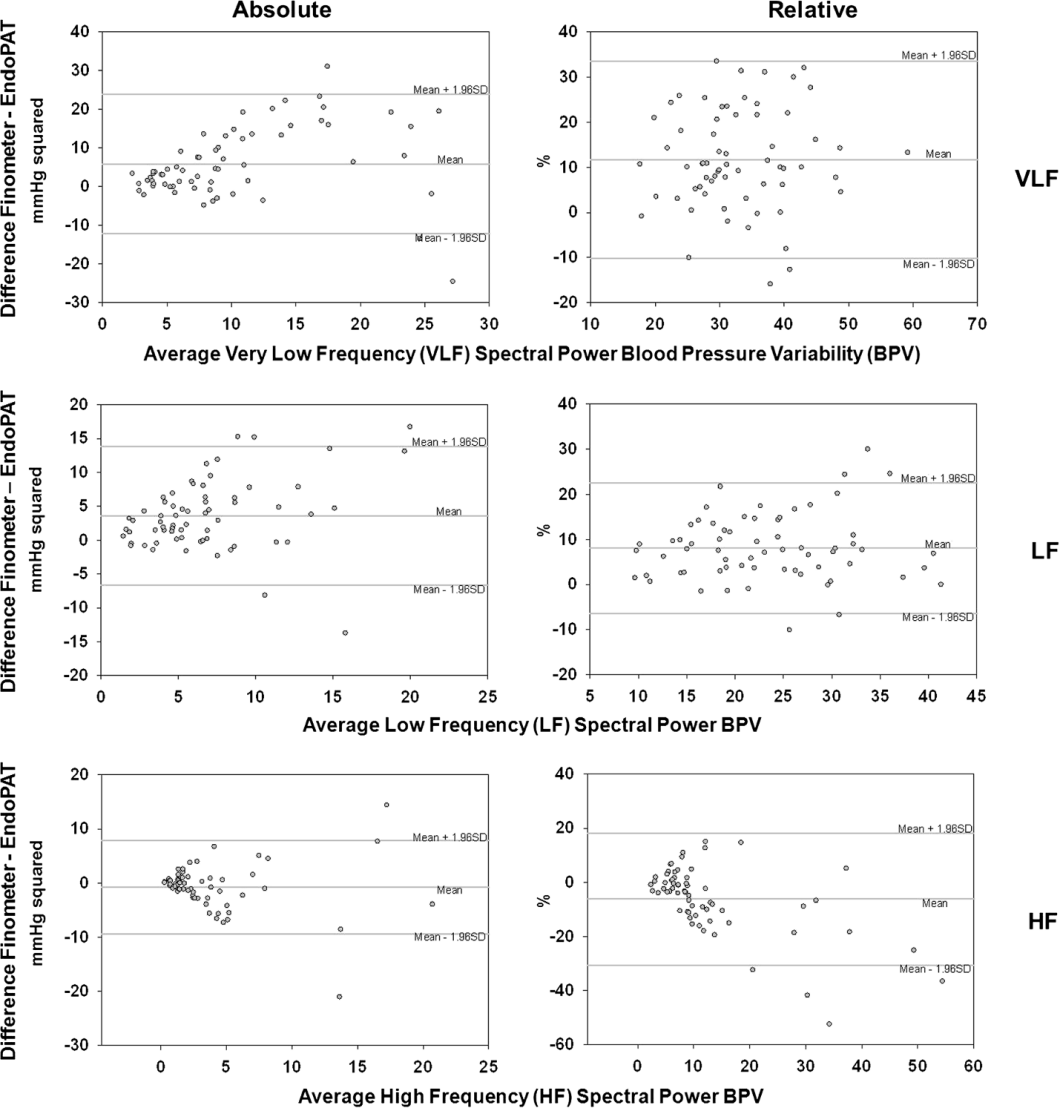
**Bland-Altman plots of spectral domain BPV parameters derived from EndoPAT and Finometer devices.** Bland-Altman comparison of frequency-domain BPV parameters between EndoPAT- and Finometer-derived time series shows low agreement between the two devices — across VLF, LF, and HF spectral ranges for both absolute and relative BPV. However, relative LF and absolute HF BPV demonstrate greater accuracy relative to other BPV measures.

Comparison by Bland-Altman plots supported the results obtained by transfer function analysis and intra-class correlation analysis, indicating that EndoPAT and Finometer provide similar results for both time - (Figure 1) and frequency-domain (Figure 2) HRV parameters, across the range of observed values. For all frequency domain BPV parameters (Figure 3), the Bland-Altman plots demonstrated poor agreement between Finometer- and EndoPAT-derived values.

## Discussion

The aim of this study was to investigate if HR and systolic BP time series derived from finger volume pulse waveforms provided by the EndoPAT device allow for reliable estimation of HRV and BPV parameters. As a validated standard, HR and systolic BP time series obtained from the Finometer device were used. To investigate if the EndoPAT-derived HR and systolic BP time series correspond to the time series derived from the Finometer device, transfer function analysis was performed. The squared coherence of the transfer function revealed excellent correlation between the HR time series derived from the two devices but indicated poor correlation for the systolic BP time series. Even without looking at the actual HRV and BPV parameters, this finding already indicates that HRV parameters may be reliably derived from waveforms provided by the EndoPAT device, while it is highly questionable if systolic BPV can be reliably assessed from the finger volume pulse waveforms. To some degree, this result was expected, as the pulse-synchronous oscillatory pattern of the finger volume pulse waveform should allow for reasonably accurate determination of inter-beat intervals. However, the waveform of the finger volume pulse is distorted relative to the standard pressure waveform provided by the Finometer device and, thus, accurate determination of beat-by-beat systolic BP may not be possible using a simple calibration procedure based on sphygmomanometer-derived BP values.

Measures of SDNN and RMSSD, as well as VLF, LF, and HF spectral HRV derived from the EndoPAT waveforms, were all in strong agreement (high ICC values) with these parameters derived from the Finometer waveforms. The ICC value for relative HF spectral power of HRV (0.77) was not as high as for VLF and LF HRV (0.94 and 0.92) but still significant. While spectral parameters of BPV derived from the EndoPAT device did not correlate well with BPV parameters derived from the Finometer, the ICC value for relative LF BPV was in the acceptable range (0.64). However, given the low LF coherence between systolic BP time series derived from EndoPAT and Finometer, this ICC value of 0.64 should be considered cautiously and may not indicate that relative LF spectral power of systolic BP can be reliably derived from EndoPAT-derived waveforms.

The ICC value for baroreflex sensitivity was even less than the ICC value for BPV. The low ICC value we observed for the baroreflex gain suggests that time series derived from the EndoPAT device do not reliably assess baroreflex function. Baroreflex analysis based on EndoPAT-derived waveforms tended to overestimate baroreflex sensitivity, and only detected approximately two thirds of the baroreflex sequences detected by Finometer-derived waveforms.

The current study is not without limitations. Both machines, to allow for simultaneous recordings, were applied to the same hand — whereas in a typical use, the equipment would be used alone on a given hand. However, this did not cause any discernible disruption in the signals. Pulses may differ across digits. Additionally, signals for both machines were not recorded in unison and were sampled at different frequencies. These design issues might contribute to discrepancies between recordings from the two machines, which if anything would bias the observed correlation to an underestimate. Thus, the correlation between measures can be assumed to be at least as strong as our study indicates. HRV from electrocardiography was not available for comparison, though HRV measures from both instruments were highly consistent with each other. The EndoPAT device includes a high-pass filter, the frequency of which is not provided, which may impact variability measures on spectral analysis, especially in the VLF range. Although the age range of our adult cohort spanned a broad range and included a balance of male and female participants, they were young and predominantly Caucasian, therefore caution should be used when extrapolating our findings to other ethnicities. While time domain parameters for HRV are generally accepted to have high inter- and intra-visit reproducibility, spectral measurements have been shown to be less reproducible between visits [19], and may thus be less useful clinically.

Even though the conclusion of this study is that parameters that depend on accurate BP waveforms, such as BPV or baroreflex function, cannot be reliably determined from the EndoPAT signal, one may speculate that it may be possible to design more advanced mathematical algorithms that may potentially be able to convert the finger volume pulse from the EndoPAT device into a BP waveform. E.g., a transfer function may be derived from the EndoPAT and Finometer recordings obtained in this study that may potentially allow for reconstruction of a more or less accurate BP waveform from the finger volume pulse signal provided by the EndoPAT device. However, such analysis is beyond the scope of this study. Similarly, our study is not applicable to the calculation of variability between routine BP readings in the clinic [20].

## Conclusions

In summary, our findings support the hypothesis that HRV can be reliably assessed by analysis of the raw EndoPAT signal (i.e., finger volume pulse waveforms), which may allow researchers to assess cardiovascular/autonomic health in addition to the manufacturer’s primary use which is to measure microvascular endothelial function. In contrast, the BP variability derived from the EndoPAT device showed low agreement with the measurements obtained from the Finometer, suggesting that the EndoPAT should not be used to assess BP variability. Still, while the EndoPAT may not be cost effective to use solely for HRV analysis, it may prove useful to employ the device for measuring HRV in subjects on which the device has already been used to assess endothelial function (i.e., retrospective analysis of existing recordings).

## Abbreviations

BP: Blood pressure
BPV: Blood pressure variability
FFT: Fast fourier transform
HF: High frequency
HRV: Heart rate variability
ICC: Intraclass correlation coefficient
LF: Low frequency
NN: Normal sinus rhythm pulse intervals
RMSSD: Root-mean-square of successive differences
SDNN: Standard eviation of NN intervals
VLF: Very low frequency.
